# Potential therapeutic targets of the nuclear division cycle 80 (NDC80) complexes genes in lung adenocarcinoma

**DOI:** 10.7150/jca.41834

**Published:** 2020-03-04

**Authors:** Zhong-Yi Sun, Wei Wang, Han Gao, Quan-Fang Chen

**Affiliations:** 1 Department of Emergency, the First Affiliated Hospital, Guangxi Medical University, Nanning, Guangxi, People's Republic of China.; 2 Institute of respiratory disease, the First Affiliated Hospital, Guangxi Medical University, Nanning, Guangxi, People's Republic of China.

**Keywords:** Lung adenocarcinoma, Prognostic, NDC80 complex, Kinetochore, Tumor

## Abstract

**Background**: Lung cancer is the most common cancer worldwide, both in terms of the incidence and mortality. NDC80 complex comprising of NDC80, NUF2, SPC24, and SPC25 is a heterotetrameric protein complex located in the outer layer of the kinetochore and plays a critical role in mitosis. This study focuses on the effects of NDC80 complex genes on clinical features and prognosis in lung adenocarcinoma (LUAD).

**Materials and methods**: Expression of NDC80 complex in LUAD and related clinical information was extracted from the TCGA website. NDC80 complex gene functional analysis and correlation analysis was conducted by using DAVID, BiNGO, Gene MANIA, STRING and GSEA. Survival probability was predicted by nomogram. Statistical analysis was used to predict NDC80 complex gene expression on clinical features and prognosis in patients with LUAD.

**Results**: Expression of NDC80, NUF2, SPC24 and SPC25 was significantly elevated in LUAD tumors compared with normal tissues (*P* < 0.05). These genes showed diagnostic values for LUAD (*P* < 0.001 for each; area under the curve (AUC), 0.958, 0.968, 0.951, and 0.932 respectively); combinatorial analysis of these genes was more advantageous than single analysis alone (*P* < 0.001; AUC > 0.900 for each). Expression of both NDC80 and SPC25 correlated with the prognosis of LUAD (*P* < 0.001; AUC > 0.600 for each). Higher expression of NDC80, NUF2, SPC24 and SPC25 was associated with low overall survival (OS) in univariate analysis. Higher expression of NDC80 and SPC25 was associated with low OS in multivariate analysis. High expression of NDC80 combined with high expression of SPC25 was predictive of poor OS in LUAD in joint analysis.

**Conclusion**: NDC80 complex gene might be an early indicator of diagnosis and prognosis of LUAD. The combined detection of NDC80, NUF2, SPC24 and SPC25 may become a new research direction in LUAD diagnosis and a new target for tumor targeted gene therapy.

## Introduction

Cancer is one of the biggest public health problems in the world 1,2. Among all cancers, lung cancer has the highest incidence and mortality rate 1,3. It is estimated that there will be 2.1 million new cases of lung cancer in 2018 and 1.8 million people will die of lung cancer, accounting for nearly one fifth of all cancer deaths (18.4%). Pathologically, lung cancer is divided into small cell lung cancer (SCLC) (15% of lung cancer cases) and non-small cell lung cancer (NSCLC) (the remaining 85% cases), which is further divided into carcinoma epidermoid of the lung cancer (LUSC) (approximately 40% of lung cancers), lung adenocarcinoma (LUAD) (approximately 20 to 30%), large cell lung cancer (approximately 15%), and undifferentiated NSCLC 2. Although rapid development of medical and clinical treatment technologies, including surgical resection, chemotherapy and targeted therapy, has saved the lives of lung cancer patients for half a century, the prognosis of patients with lung cancer is still not optimistic - the diagnosis rate for advanced lung cancer is approximately 80% and the average survival rate at 5 years is only 15% 2, 4.

With the deterioration of the natural environment, non-smoking lung cancer and lung cancer in women has increased sharply in recent years. The prevalence of LUAD is gradually surpassing that of lung squamous cell carcinoma (LUSC) 5. Lack of biomarkers of early diagnosis due to the occultation process, about 50% of patients with at the time of LUAD diagnosis has local infiltration and distant metastasis. The 5-year survival rate of clinical-stage 4 patients is less than 1% 3, 5, 6.

Factors responsible for lung cancer include genetic and signaling pathway abnormalities. Hence, it is important to understand the associated genes and their mechanisms in the development of lung cancer 3, 7, 8. The nuclear division cycle 80 (NDC80) complexes consisting of NDC80, NUF2, SPC24, and SPC25 form a heterotetrameric protein complex located in the outer layer of the kinetochore and link the kinetochore to microtubules during mitosis 9-11. Abnormal production of any of the NDC80 complex genes can cause chromosomal aberration and instability of the genome a major event in all tumorigenesis 12. Studies have shown aberrant expression of the NDC80 complex in various tumors, which can be used as a diagnostic marker for certain tumors, and may even be an indicator for evaluating prognosis 13-15. However, the role of the NDC80 complex in LUAD is not very clear. In this study, we studied the effects of NDC80 complex genes on clinical characteristics and prognosis in LUAD.

## Method and Materials

### Source of patient data

Expression of the NDC80 complex in a total of 500 LUAD patients along with clinical information including age, sex, smoking history, radiation therapy history, targeted therapy history, neoplasm status, TNM stage and residual tumors were extracted from The Cancer Genome Atlas (TCGA: https://cancergenome.nih.gov/ October 2, 2019) and University of California Santa Cruz Xena (UCSC Xena: https://xena.ucsc.edu/. October 2, 2019). Boxplots of NDC80 complex expression in normal and tumor tissues were created through Gene Expression Profiling Interactive Analysis (GEPIA, http://gepia.cancerpku.cn/, October 13, 2018) 16. Patients with missing overall survival (OS) status, OS time, and/ or missing expression data, were excluded. Only the first test data was included from patients with repeated expression data.

### NDC80 complex functional and correlation analysis

A Pearson correlation matrix to understand the correlation among the NDC80 complexes genes was constructed using R version 3.6.1 (https://www.r-project.org/, October 2, 2019). Functional and enrichment analysis using the Database for Annotation, Visualization, and Integrated Discovery (DAVID) v.6.8 (https://david.ncifcrf.gov/tools.jsp, October 2, 2019) 17, 18, including functional analysis of gene ontology (GO) and analysis of the Kyoto Encyclopedia of Genes and Genomes (KEGG) pathway. GO functional analysis included biological process (BP), molecular function (MF), and cellular component (CC). The function of the gene was predicted using the GO function analysis tool Biological Networks Gene Ontology (BiNGO) based on the results of the correlation analysis 19. Interaction between the members of the NDC80 complex was analyzed by gene function prediction on Gene MANIA (Gene MANIA: http://genemania.org/, October 2, 2019) 20. The Search Tool for the Retrieval of Interacting Genes/Proteins (STRING: http://string-db.org, October 12, 2019) was used to evaluate the functional and physical relationships of NDC80 complex and correlated genes 21.

### Diagnostic and prognostic analysis

Diagnostic receiver operating characteristic (ROC) curves were constructed using the mRNA expression of NDC80 complex genes in tumor and non‑tumor tissues 22, 23.

### Analysis of survival

Patients were subdivided into low- and high-expression groups according to the median OS. OS was used to evaluate prognosis of LUAD. Correlation among the NDC80 complex genes was identified by Kaplan-Meier estimator with a log-rank test. Values were adjusted for age, sex, smoking history, radiation therapy history, targeted therapy history, neoplasm status, TNM stage and residual tumors in the Cox proportional hazards regression model. The effect of high and low expression of each gene of the NDC80 complex on the prognosis was also evaluated.

### Joint-effects survival analysis

Joint effect analysis was performed on genes with significant differences (*P* < 0.05) in OS. NDC80 complex genes with prognostic value in multivariate survival analysis were grouped as better OS, worse OS, or other. Log-rank test and Kaplan-Meier analysis was used to evaluate the prognostic value of the NDC80 complex in each group.

### Nomogram construction

A prognostic risk score was based on the adjusted expression levels (TNM stage, neoplasm status, residual tumor, radiation therapy) of NDC80 and SPC25 in LUAD. 1-year, 3-year, and 5-year survival rates were predicted based on clinical factors and genes that were used to construct the Nomogram for OS 24.

### Gene set enrichment analysis (GSEA)

Relationship between the NDC80 complex gene expression and OS in LUAD patients was explored by GSEA. Pathway-based analysis in LUAD with high and low expression of each of the NDC80 complex genes was performed by comparing the reference c5 (GO gene sets: c5.all.v6.1.symbols.gmt) and c2 (KEGG gene sets: c2.all.v6.1.symbols.gmt) gene sets from Molecular Signatures Database (MSigDB) using GSEA v.4.0.1 (http://software.broadinstitute.org/gsea/msigdb/in dex.jsp, October 2, 2019) 25.

### Statistical analysis

Statistical analysis was performed with SPSS v.22.0 software (IBM, Chicago, IL, USA). Vertical scatter plots and survival curves were generated in GraphPad Prism v.8.0 (GraphPad Software, La Jolla, CA, USA) and R 3.6.1 (http://www.R-project.org). OS was analyzed by Kaplan-Meier curve and log-rank test. Multivariate survival analysis was evaluated with hazard ratios (HR), and 95% confidence intervals (CIs) were calculated using Cox proportional hazards regression with adjustment for influential clinical characteristics, including age and tumor stage. P < 0.05 was considered statistically significant.

## Results

### Clinical characteristics of patients

**Figure 1:**
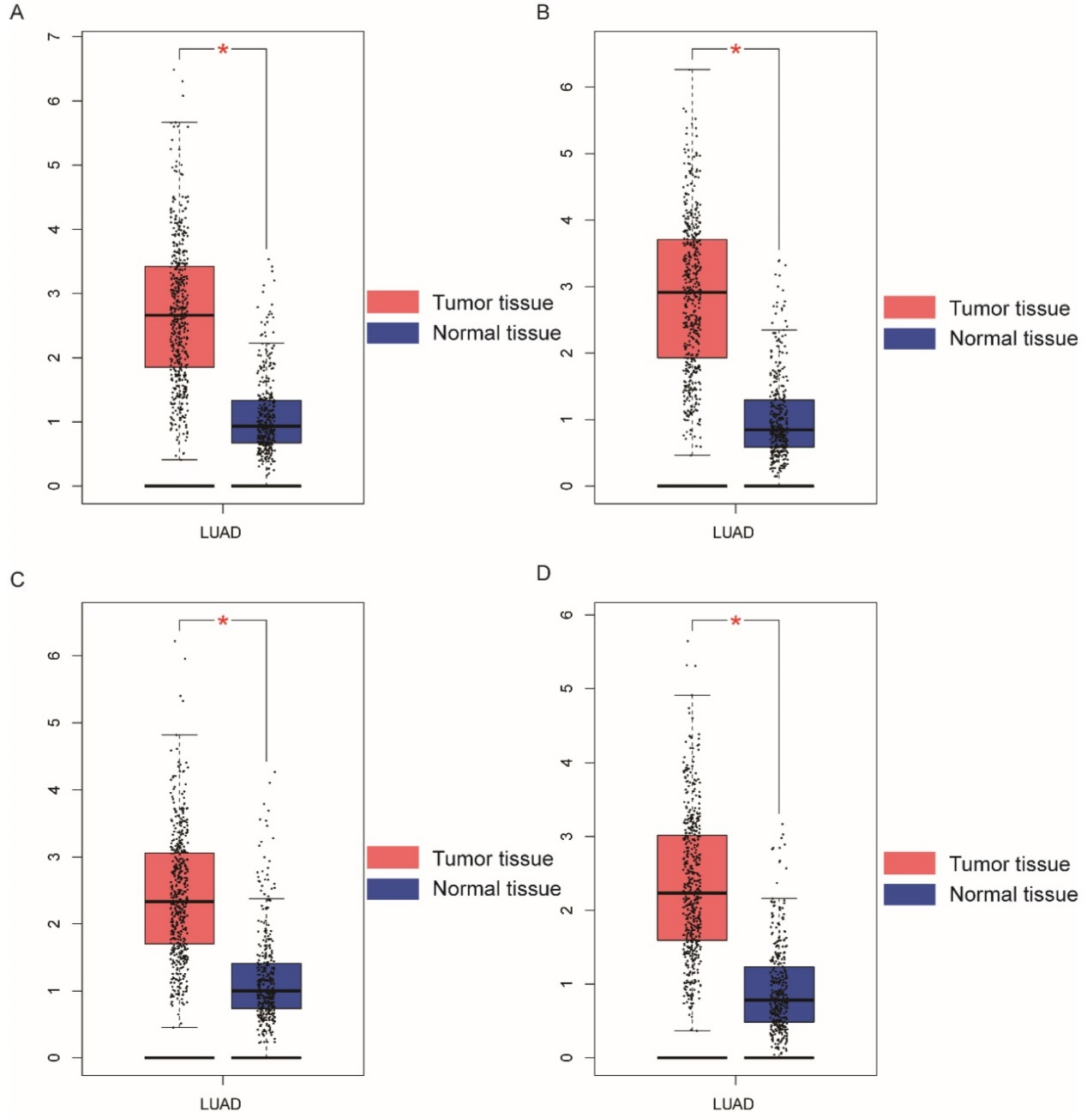
** Boxplots showing NDC80 complex gene expression levels in LUAD and normal tissue.** (A) NDC80; (B) NUF2; (C) SPC24; (D) SPC25; Abbreviations: NDC80 complex, nuclear division cycle 80 complex, GEPIA, gene expression profiling interactive analysis. *P < 0.05.

**Figure 2:**
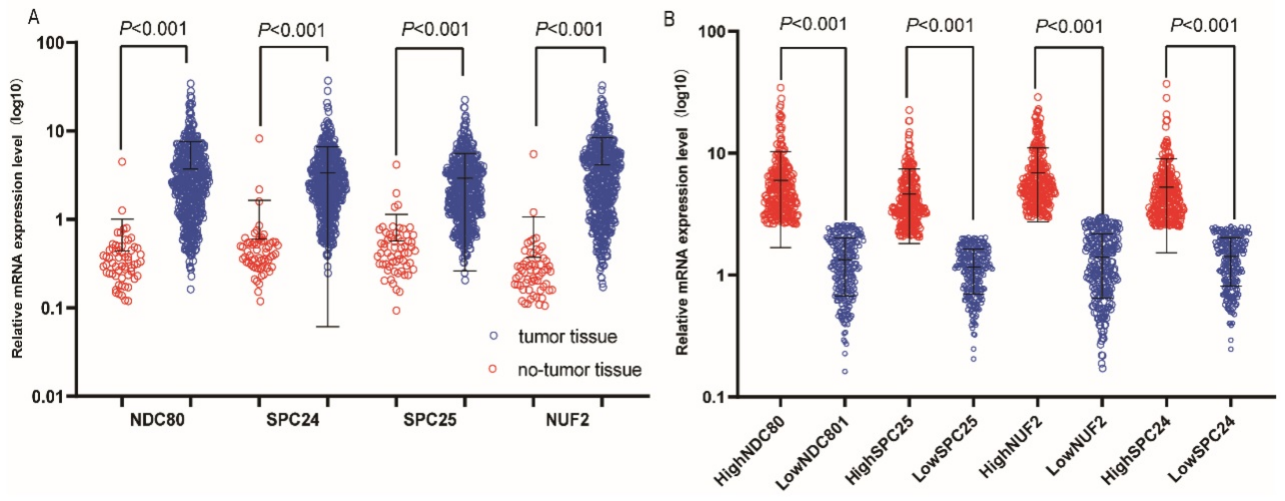
** Relative mRNA expressions of NDC80 complex in tumor and normal tissues and low, high expression groups.** (A) Relative mRNA expressions of NDC80 complex gene in tumor and normal tissues; (B) Relative mRNA expressions of NDC80 complex in low and high expression groups. NDC80 complex, nuclear division cycle 80 complex.*P<0.05.

Demographic characteristics, clinical features, and relationship to OS in patients with LUAD are presented in **Table 1**. All clinical data and demographics of were obtained from TCGA.TNM stage; neoplasm status, residual tumor, and radiation therapy were associated with OS (*P* < 0.001, respectively). Boxplots of NDC80 complex in normal and tumor tissue are presented in **Figure 1**; Expression of NDC80, NUF2, SPC24, and SPC25 were significantly higher in LUAD than healthy lungs** (Figure 2A). Figure 2B** shows the level of stratified expression of the NDC80 complex genes in LUAD.

### Correlation, function and bioinformatics analysis

**Figure 3:**
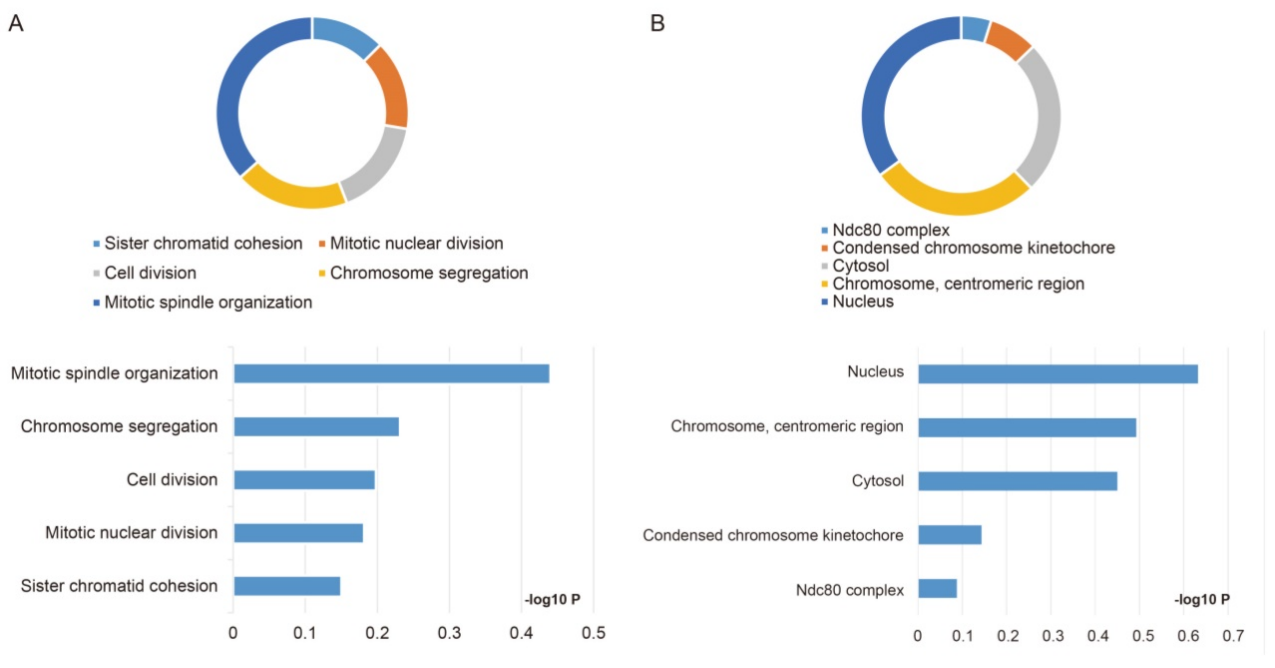
** Outcomes of GO analysis of functional enrichment assessed by DAVID:** (A) BP outcomes; (B) CC outcomes. Abbreviations: BP, biological process; CC, cellular component.

**Figure 4:**
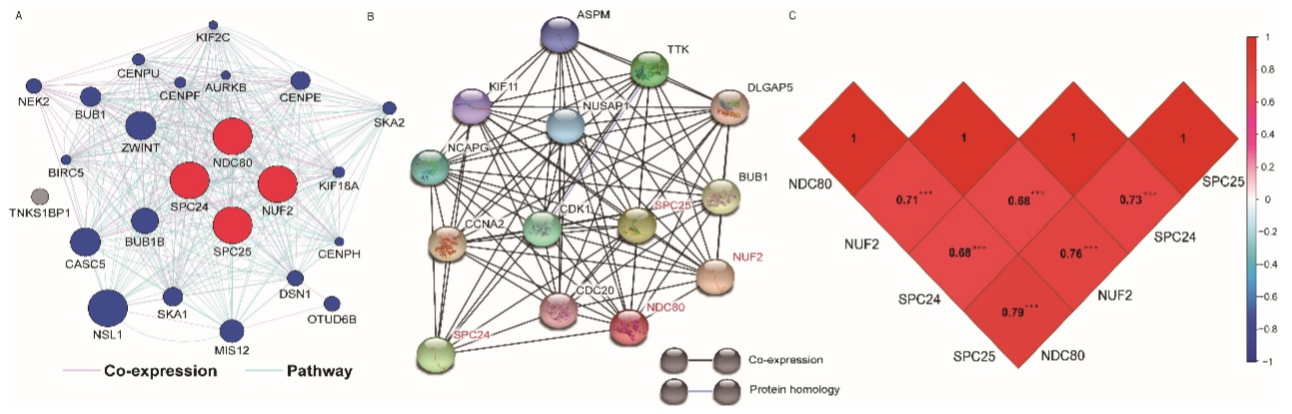
(A) Gene interaction networks among selected genes generated by GeneMANIA; (B) STRING physical and functional connections of NDC80 complex gene (C) Pearson's correlation coefficients for NDC80, NUF2, SPC24 and SPC25 gene expression levels.

**Figure 5:**
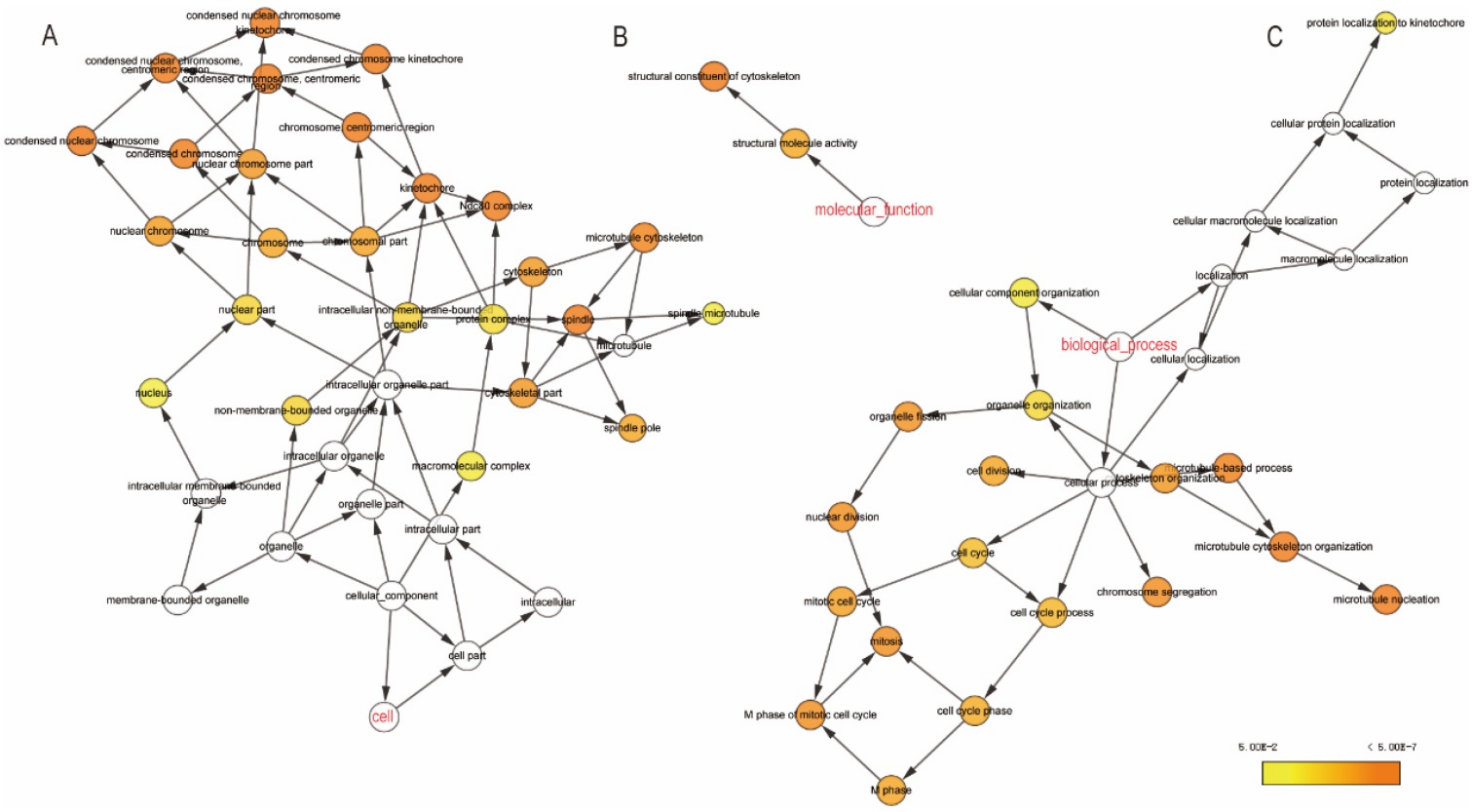
(A) CC outcomes; (B) MF outcomes; (C) BP outcomes of GO analysis of functional enrichment by BiNGO. Abbreviations: CC, cellular component; MF, molecular function; BP, biological process; Biological Networks Gene Ontology.

The GO function and KEGG pathway examination through DAVID showed that NDC80 complex genes is closely related to mitotic spindle organization, chromosome segregation, cytosol et al. (**Figure 3A,B**); Gene‑gene co‑expression interactions and pathway prediction among NDC80 complex genes is shown in **Figure 4A.** The integration method for examining protein-protein co-expression by STRING is illustrated in **Figure 4B**. Association between NDC80, NUF2, SPC24 and SPC25 is shown in Pearson correlation matrix (**Figure 4C**). Expression of the NDC80 complex genes significantly correlated with each other *P* < 0.001. Results of co-functional analysis using BiNGO indicated that the NDC80 complex genes correlated with cell division, mitotic spindle organization, mitotic nuclear division, chromosome segregation and sister chromatid cohesion (**Figure 5**).

### Diagnostic and prognostic value of NDC80 complex genes

**Figure 6:**
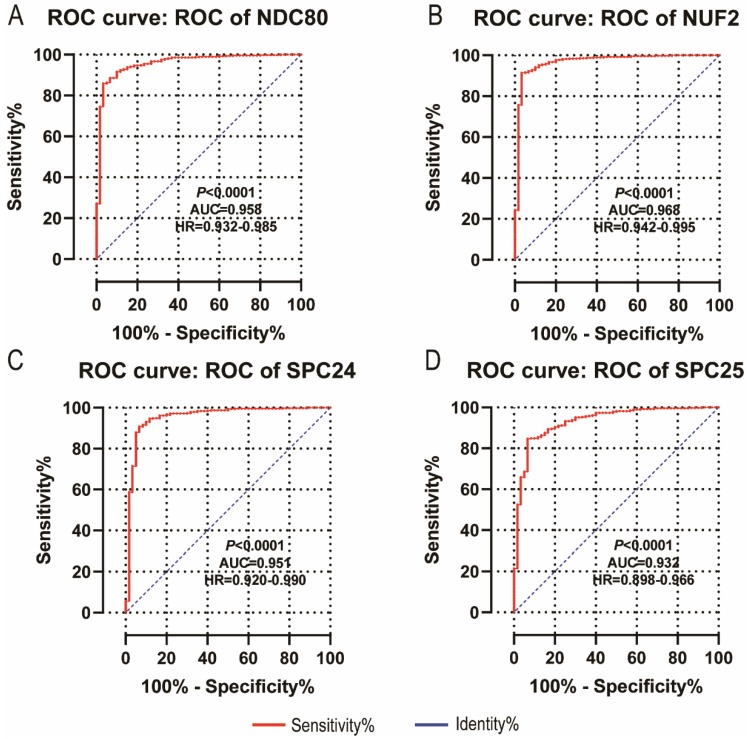
** Diagnostic ROC curves of NDC80, NUF2, SPC24 and SPC25.** In particular, diagnostic ROC curves of (A) NDC80, (B) NUF2, (C) SPC24, (D) SPC25. Abbreviations: ROC, receiver operating characteristics; AUC, area under the curve; CI, confidence interval.

**Figure 7:**
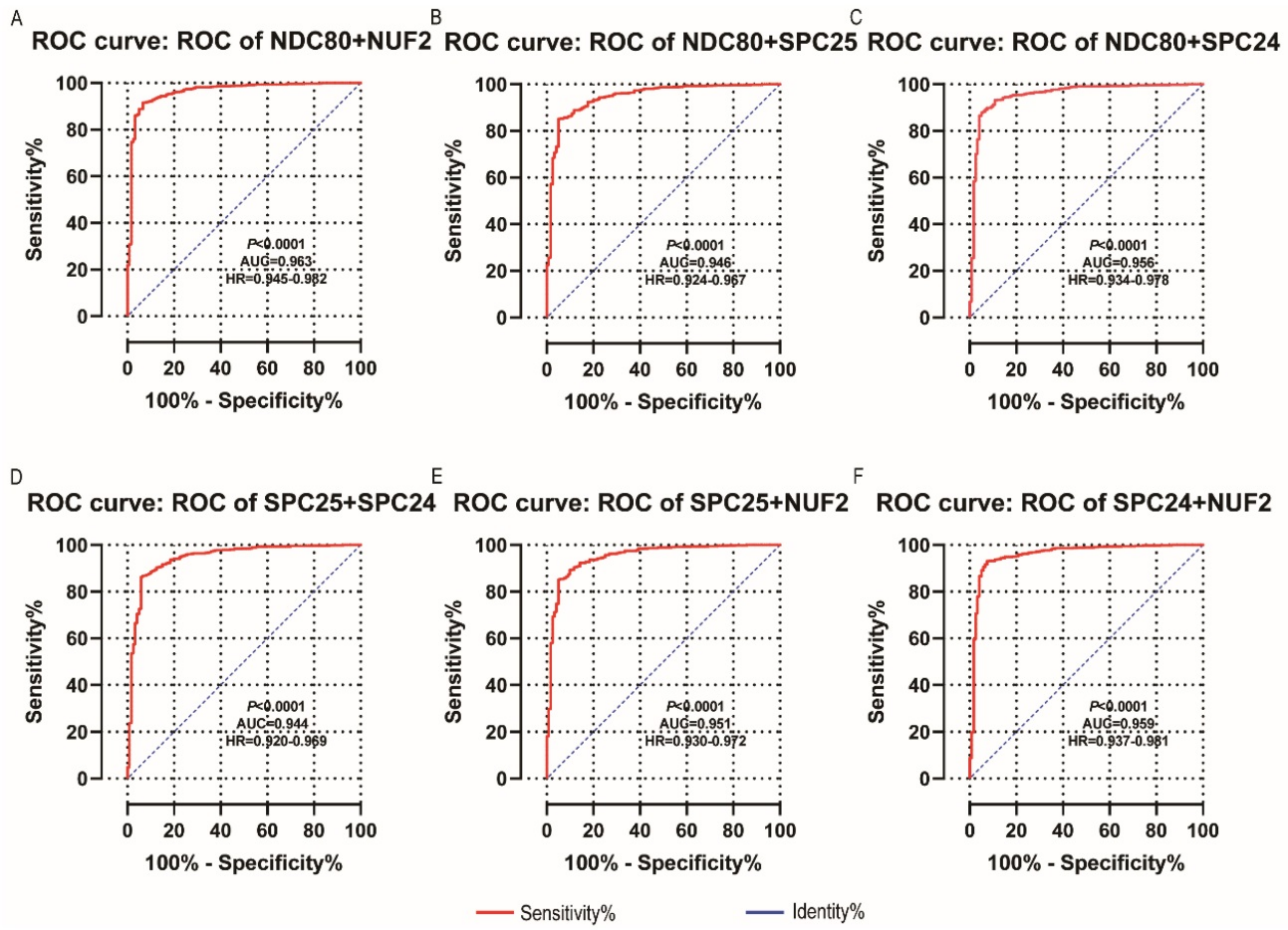
** Diagnostic ROC curves of combination of:** (A) NDC80 + NUF2, (B) NDC80 + SPC25, (C) NDC80 + SPC24, (D) SPC24 + SPC25, (E) SPC25 + NUF2, (F) SPC24 + NUF2. Abbreviations: ROC, receiver operating characteristics; AUC, area under the curve; CI, confidence interval.

**Figure 8:**
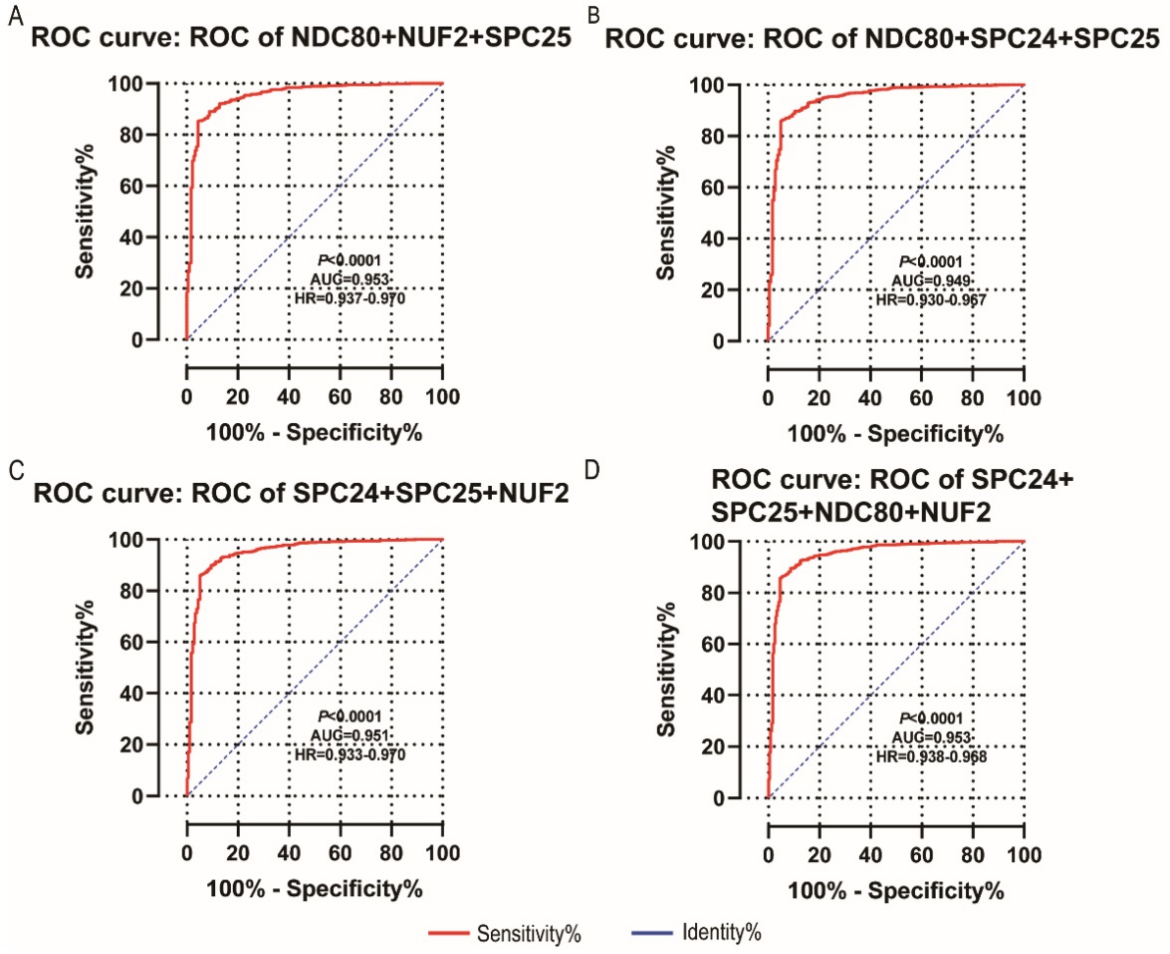
** Diagnostic ROC curves of combination of:** (A) NDC80 + NUF2 + SPC25, (B) NDC80 + SPC24 + SPC25, (C) SPC24 + SPC25 + NUF2, (D) SPC24 + SPC25 + NDC80 + NUF2. Abbreviations: ROC, receiver operating characteristics; AUC, area under the curve; CI, confidence interval.

**Figure 9:**
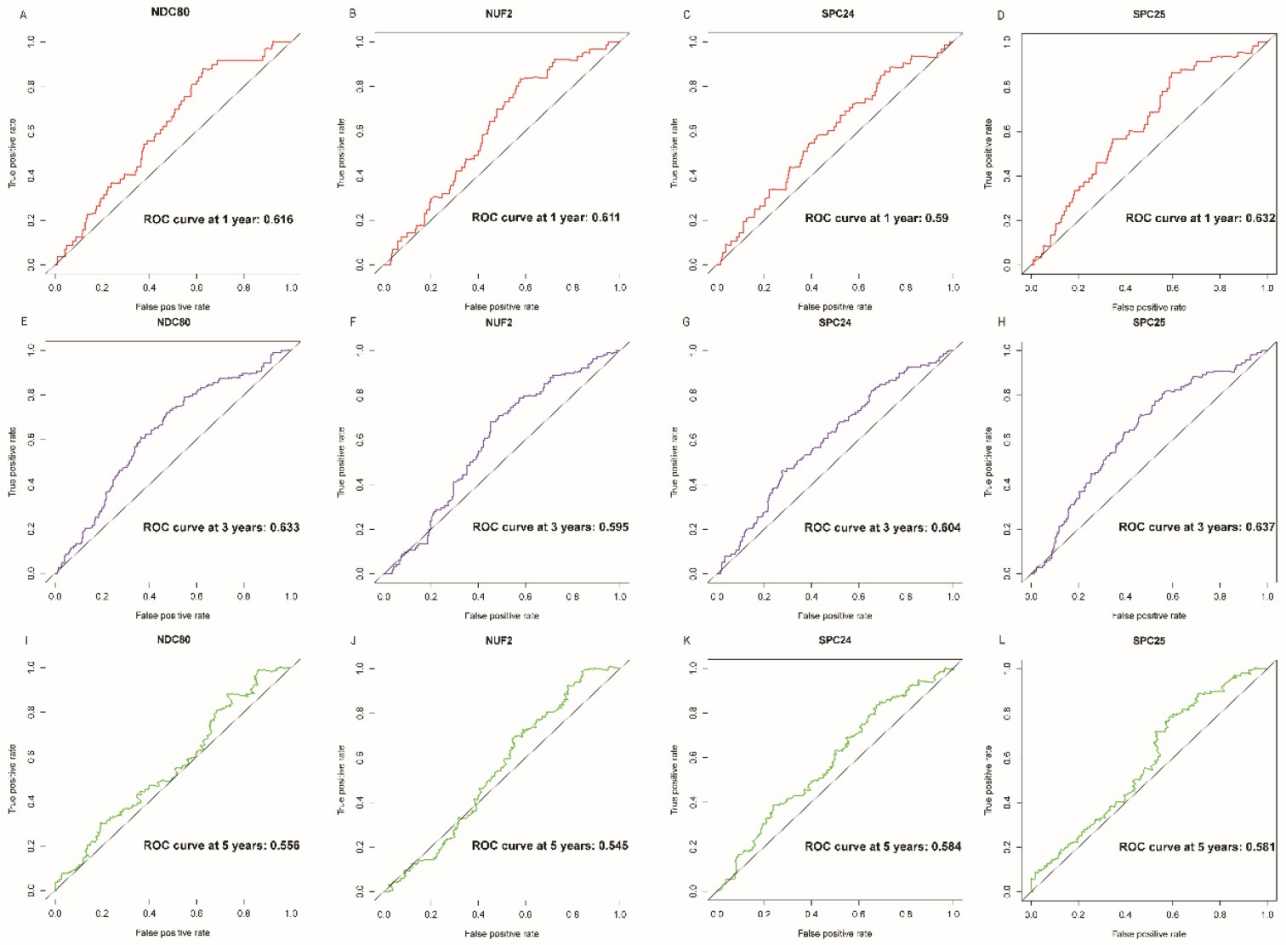
** Overall survival ROC curves of NDC80 complex gene at 1, 3 and 5 years. ROC curves of:** (A) NDC80, (B) NUF2, (C) SPC24, and (D) SPC25 at 1 year; (E) NDC80, (F) NUF2, (G) SPC24, and (H) SPC25at 3 years; (I) NDC80, (J) NUF2, (K) SPC24, and (L) SPC25at 5 years. Abbreviation: ROC, receiver operating characteristics.

NDC80, NUF2, SPC24 and SPC25 showed diagnostic value for LUAD (*P* < 0.001for each; area under the curve (AUC) was 0.958, 0.968, 0.951, 0.932 respectively;** Figure 6A - 6D**). Combinations of NDC80 + NUF2, NDC80 + SPC25, NDC80 + SPC24, SPC24 + SPC25, SPC25 + NUF2, SPC24 + NUF2, NDC80 + NUF2+ SPC25, NDC80 + SPC24 + SPC25, SPC24 + SPC25 + NUF2, SPC24 + SPC25 + NDC80 + NUF2 also showed diagnostic value for LUAD (*P* < 0.001for each; AUC was 0.963, 0.946, 0.956, 0.944, 0.951, 0.959, 0.953, 0.949, 0.951, 0.953, respectively; **Figure 7A - 7F** and **Figure 8A - 8D**). Both NDC80 (all AUC >0.600; **Figure 9A, E**) and SPC25 were associated with OS at 1‑ and 3‑ year OS (all AUC >0.600; **Figure 9D, H**).

### Survival analysis

**Figure 10:**
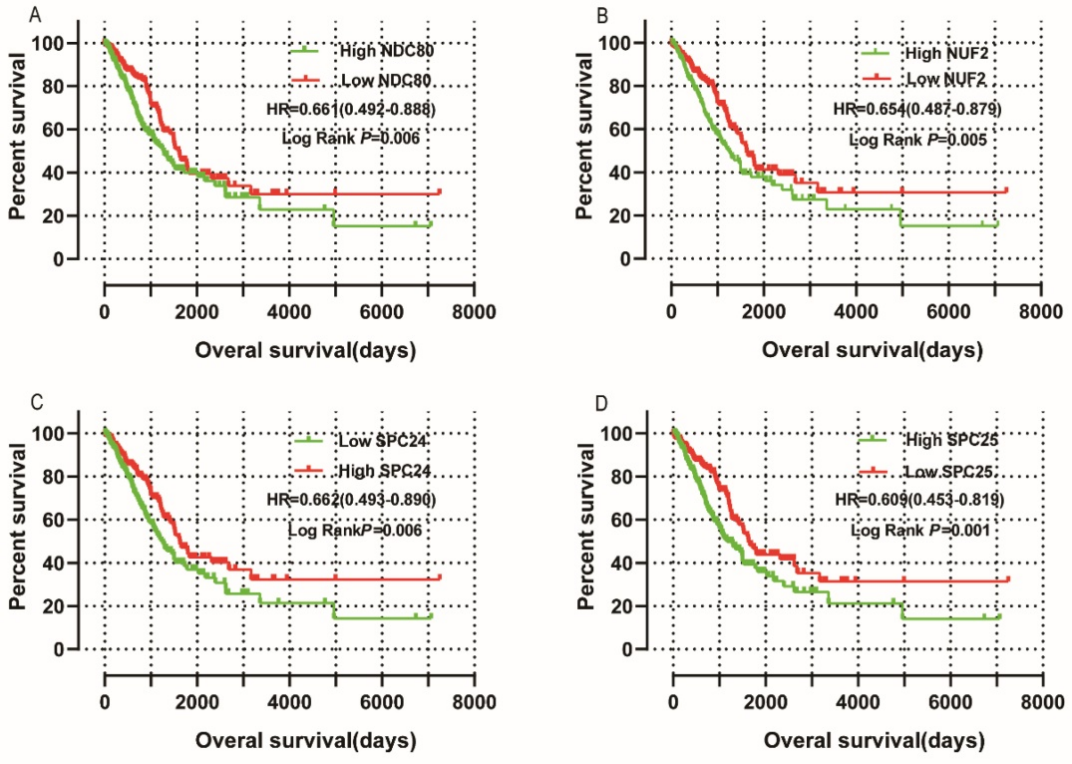
** Univariate survival analyses:** (A) NDC80, (B) NUF2, (C) SPC24, and(D) SPC25 (n=500).

Survival analysis is shown in** Figure 10** and summarized in** Table 2.** Low mRNA expression of NDC80, NUF2, SPC24, SPC25 in LUAD was related to favorable OS in univariate survival analysis (log-rank *P =* 0.006, HR = 0.661, 95%CI = 0.492 - 0.888;** Figure 10A;** log-rank *P =* 0.005, HR = 0.654, 95%CI = 0.487 - 0.879;** Figure 10B;** log-rank *P =* 0.006, HR = 0.662, 95%CI = 0.493 - 0.890;** Figure 10C;** log-rank *P =* 0.001, HR = 0.609, 95%CI = 0.453 - 0.819;** Figure 10D,** respectively). Low expression of NDC80 and SPC25 was also associated with favorable OS in multivariate analysis when adjusted for radiation therapy history, targeted therapy history, neoplasm status, TNM stage and residual tumors (log-rank *P =* 0.032, HR=0.635, 95%CI = 0.419 - 0.962; log-rank *P =* 0.047, HR=0.635, 95%CI = 0.434 - 0.995, respectively).

### Joint‑effect survival analysis

**Figure 11:**
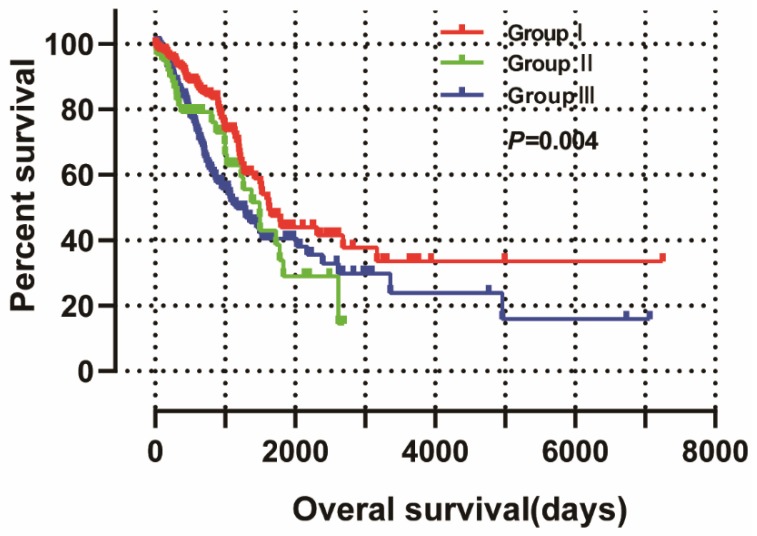
Joint-effects survival analysis of the influence of combined NDC80 complex gene expression on OS stratified for NDC80 and SPC25 expression levels.

The joint‑effect survival analysis was based on multivariate survival analysis and was used to reveal the combined effects of NDC80 and SPC25 on OS in LUAD. Patients are grouped by expression level as shown in **Table 3** and the results of the group are shown in **Table 4** and **Figure 11**. Low expression of NDC80 and SPC25 in group I was tied to favorable OS (*P* < 0.05). However, high expression of NDC80 and SPC25 in group III was tied to unfavorable OS (*P* < 0.05).

### Risk score model of nomogram

**Figure 12:**
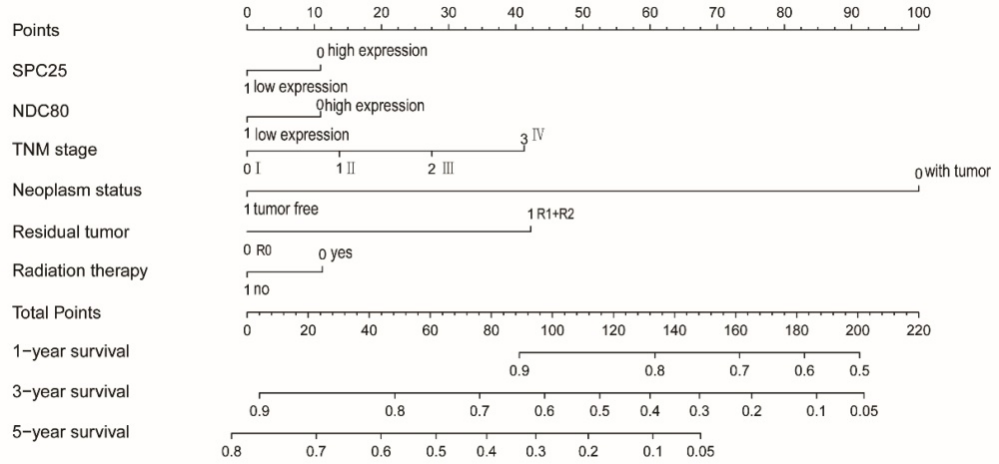
Nomograms constructed using overall survival and recurrence-free survival-related clinical factors and genes.

NDC80 and SPC25 expression, TNM grade, tumor status, residual tumors, radiotherapy were used to construct a nomogram for risk assessment. Points were assigned to each variable based on the Cox regression coefficients. Add these points and draw a vertical line between the total point axis and the survival probability axes at 1 year, 3 years and 5 years to estimate the probability of survival (**Figure 12**).

### Gene set enrichment analysis (GSEA)

**Figure 13:**
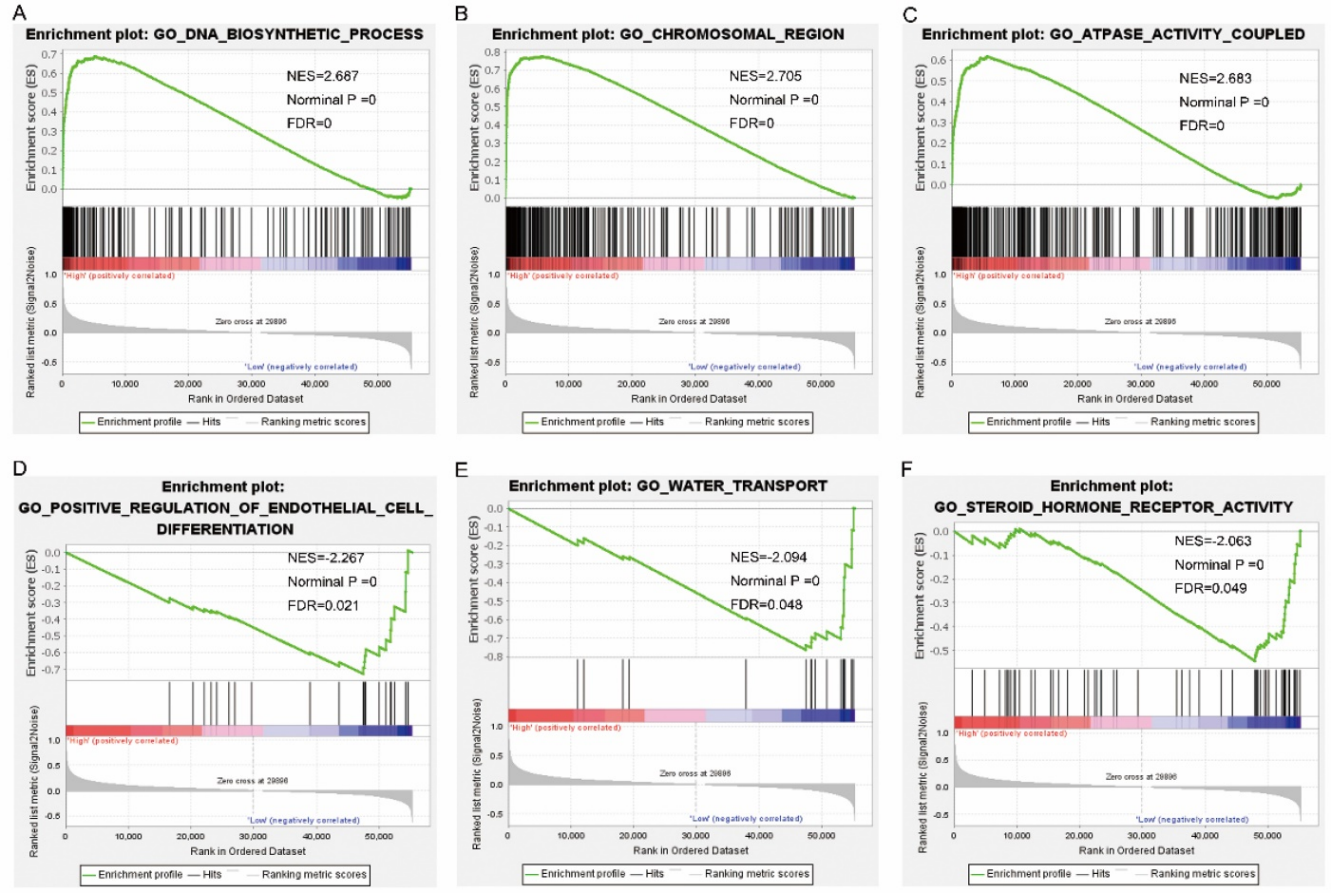
Gene set enrichment analysis results of nuclear division cycle 80. Results of gene ontologies: (A) DNA biosynthetic process; (B) chromosomal region; (C) ATPase activity coupled; (D) positive regulation of endothelial cell differentiation; (E) water transport; (F) steroid hormone receptor activity. Abbreviations: GO, gene ontology; NES, normalized enrichment score; FDR, false discovery rate.

**Figure 14:**
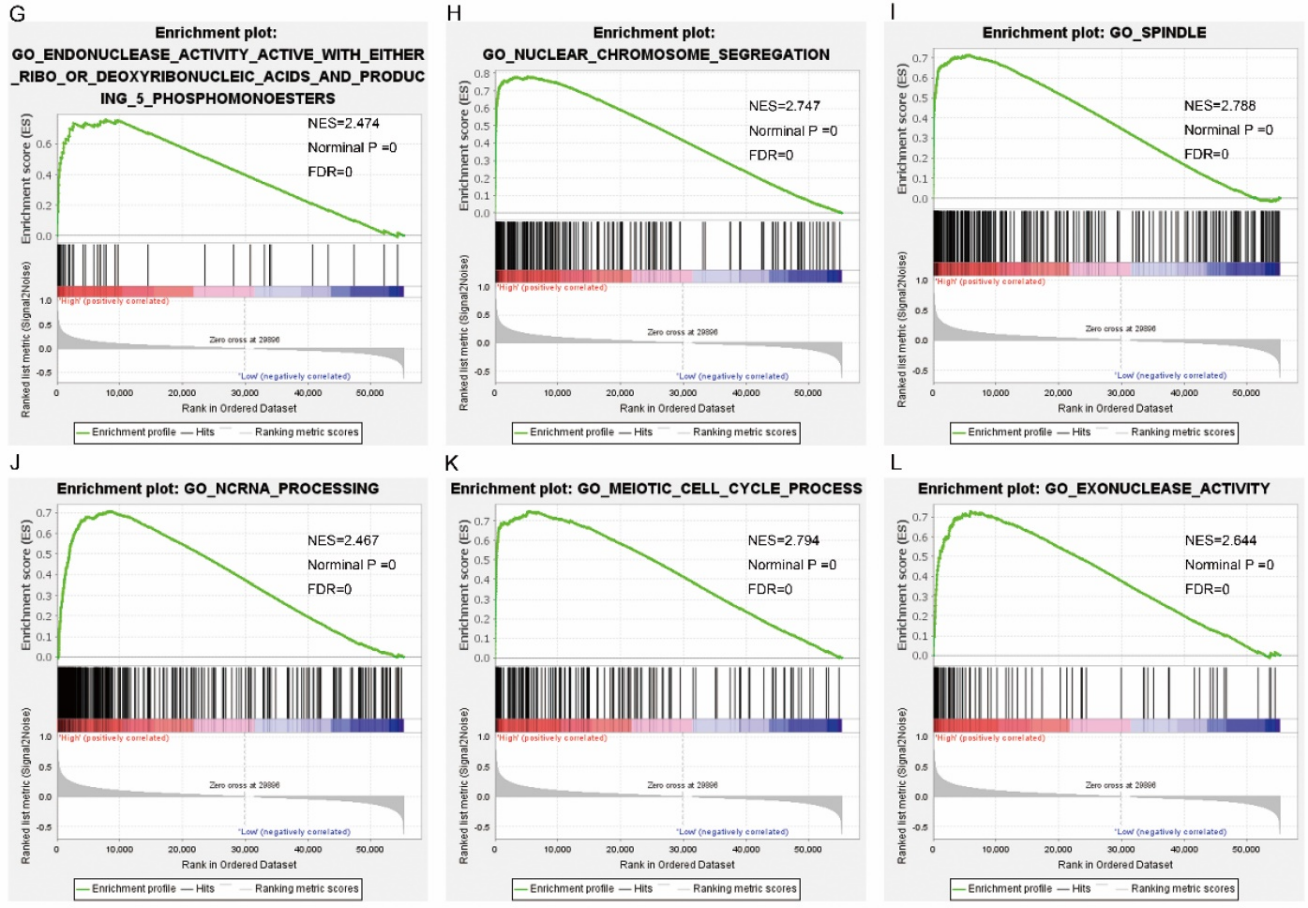
Gene set enrichment analysis results of nuclear division cycle 80. Results of gene ontologies: (G) endonuclease activity; (H) nuclear chromosome segregation; (I) spindle; (J) ncRNA processing; (K) meiotic cell cycle; (L) exonuclease activity. Abbreviations: GO, gene ontology; NES, normalized enrichment score; FDR, false discovery rate.

**Figure 15:**
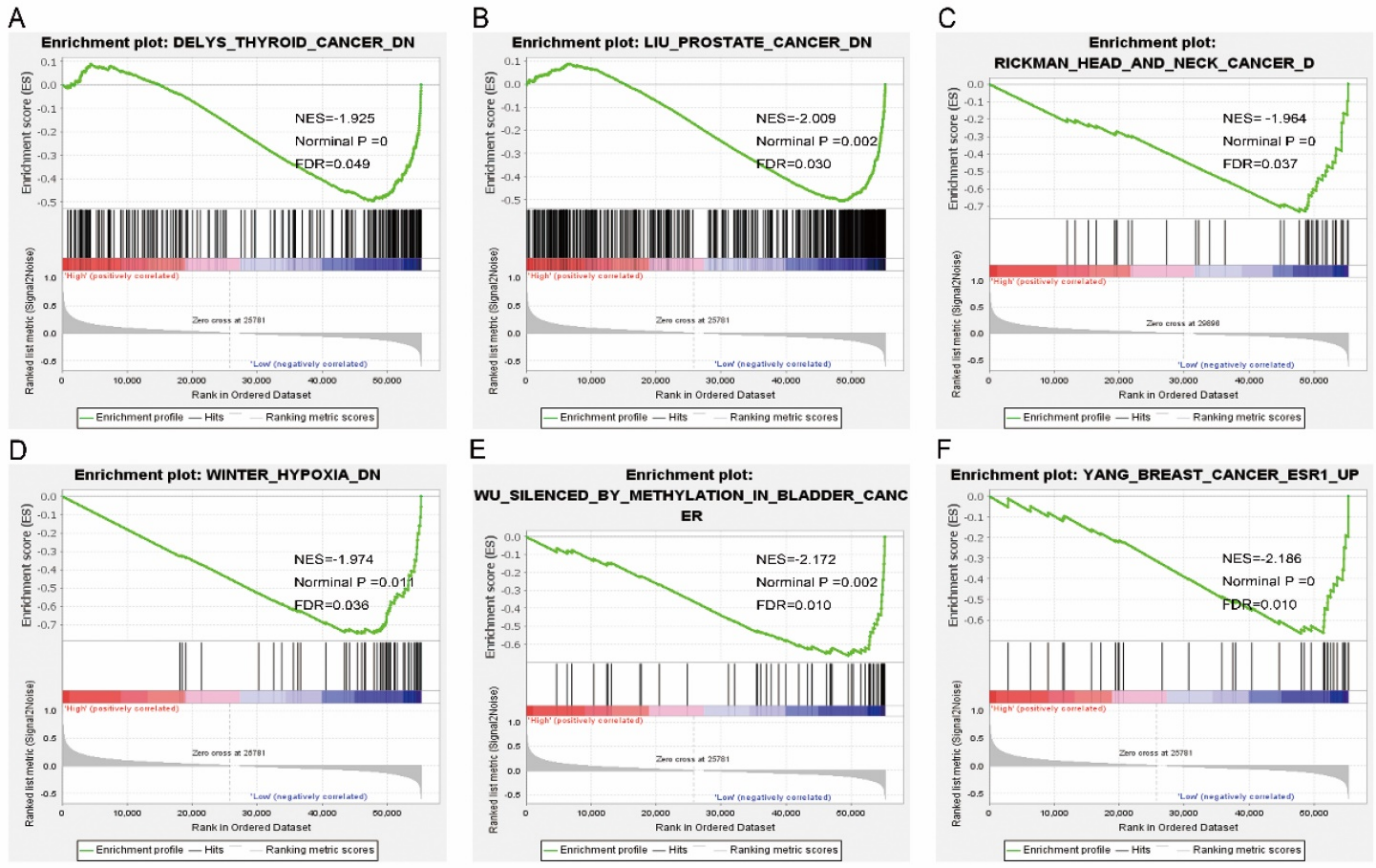
** Gene set enrichment analysis results of SPC25. Results of gene ontologies:** (A) thyroid cancer; (B) prostate cancer; (C) head and neck cancer; (D) winter hypoxia; (E) bladder cancer; (F) breast cancer Abbreviations: NES, normalized enrichment score; FDR, false discovery rate.

**Figure 16:**
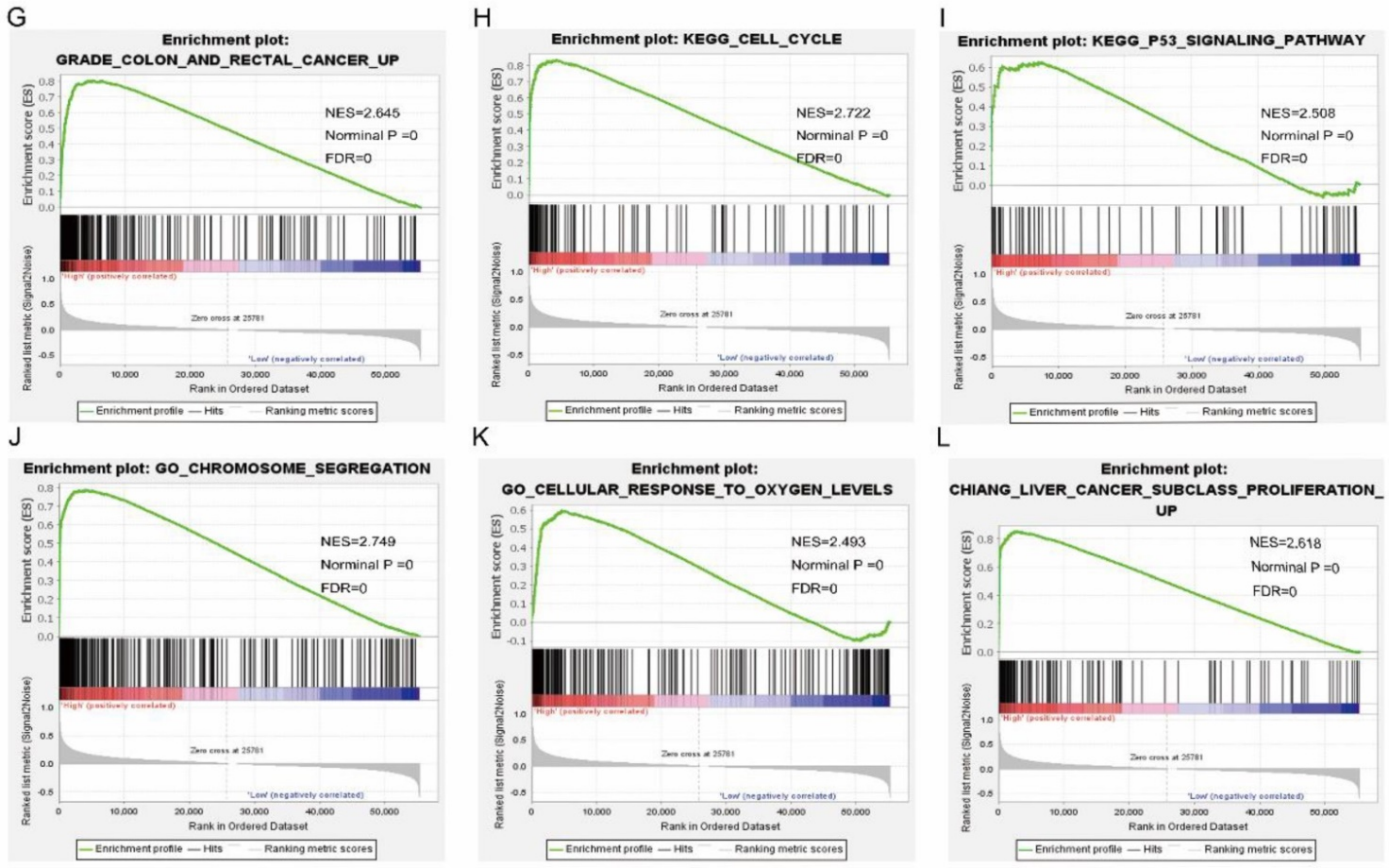
Gene set enrichment analysis results of SPC25. Results of gene ontologies: (G) colon and rectal cancer; (H) cell cycle; (I) p53 signaling pathway; (J) chromosome segregation; (K) cellular response to oxygen levels; (L) liver cancer subclass proliferation up. Abbreviations: NES, normalized enrichment score; FDR, false discovery rate.

Pathway analysis of high and low expression of each of the NDC80 complex genes showed that GO terms and KEGG pathways associated with NCD80 included among others, ATPase activity, cell cycle, water transport, chromosomal region, nuclear chromosome segregation, cell differentiation, and DNA biosynthetic process (**Figure 13 (A-D)**), **Figure 14 (A-D)**). The enriched GO terms and KEGG pathways associated with SPC25 included among others, cell cycle, bladder cancer, prostate cancer, thyroid cancer, Rickman head and neck cancer, breast cancer, oxygen levels, colon and rectal cancer, p53 pathway (**Figure 15 (A-D)**,** Figure 16 (A-D)**). The details of the results are shown in Supplementary **Tables 1** and **2**.

## Discussion

In the current study, we studied the relationship between gene expressions of the members of NDC80 complex in LUAD in the TGCA database. A risk assessment model including clinical factors and gene expression was developed to assess the diagnostic and prognostic values in LUAD patients. The function of the NDC80 complex and associated genes in LUAD was predicted. Lower expression of NDC80 complex genes was associated with good OS and expression of NDC80 and SPC25 showed diagnostic and prognostic value in LUAD. Expression of NDC80, NUF2, SPC24 and SPC25 was found significantly higher in LUAD than normal tissue. In addition, NDC80, NUF2, SPC24 and SPC25 showed diagnostic value for LUAD. Combination of NDC80 with the other genes showed diagnostic advantage over NDC80, NUF2, SPC24 and SPC25 alone suggesting that the patient expressing more than one NDC80 complex genes would have more chance to get LUAD. Overall survival ROC curves and nomograms showed that expression of NDC80, SPC25 was associated with OS. GO term analysis, protein-protein interaction (PPI) analysis, and KEGG analysis predicted the function among NDC80 complexes genes and NDC80 complexes genes correlated genes. The result showed that NDC80 complexes play important roles in cell division, mitotic spindle organization, mitotic nuclear division, and chromosome segregation and sister chromatid cohesion.

The role of NDC80 in cancer is well described. Studies have shown that overexpression of NDC80 can result in permanent hyper activation of mitotic control points and induce tumor formation in vivo 36. By constructing a high-expression NDC80 mouse model and a non-transgenic murine model, Sotillo R et al. reported that over-expression of NDC80 resulted in higher incidences of liver and lung cancer in mice 37. This was observed in conjunction with elevated expression of* Mad2* 37. Expression of NDC80 mRNA was also reported to be elevated in both gastric and pancreatic cancers 38, 39. In osteosarcoma, 84.6% of tumor tissues expressed NDC80 mRNA higher than adjacent normal tissues, and expression level correlated with tumor TNM stage and distant metastases, and NDC80 was an independent prognostic indicator 40. Expression of NDC80 protein in colon cancer cell lines such as HCT8, SW480, CACO2 and HCT116 was superior to that of a normal intestinal epithelial cell line NCM460 41. Cell proliferation was significantly accelerated after staining with the NDC80 gene and shows greater transfer capacity 41. Previous studies have shown that in vitro culturing of hepG2 hepatoma cell lines resulted in decreased NUF2 expression and cell cycle proteins such as, cyclins B1, Cdc25A and Cdc2, but expression of apoptosis-associated proteins (such as Bad and Bax) was significantly increased, thus inducing cells, inhibiting cell cycle and apoptosis, thereby inhibiting cell growth. HepG2 cells with NUF2 gene knockout were injected into the right abdomen of nude mice and the growth rate was significantly lower than non-transgenic knockout cells, indicating that the NUF2 plays an important role in the growth of liver cancer cells in vitro and in vivo 42, 43. Juan Zhou et al. reported that SPC24 regulates PI3K/AKT kinase pathway and the knockdown of SPC24 can lead to attenuated cell growth, increased cell apoptosis and cell cycle progression 44. In LUAD, previous study also found SPC24 is strongly expressed in LUAD and its level of expression is related to the survival rate for lung cancer patients. High expression of SPC24 can negatively regulate E-cadherin, and positively regulate N-cadherin and vimentin and participation in epithelial-mesenchymal transition during lung cancer, affecting tumor growth and invasion 44. In addition, high expression of SPC24 is also found in thyroid cancer, liver cancer, and osteosarcoma 44-46. However, the role SPC25 in cancer remains understudied. SPC25 is highly expressed in the basal part of breast cancer with more stem cell-like cells, and SPC25 expression is related to disease-free survival. Expression of SPC25 is higher in CpG Island methylation phenotype positive kidney carcinoma (CIMP) than in CIMP negative kidney cancer cells, but the significance remains uncertain 47.

This study had some limitations. First, the sampling size was small. For better accuracy and validation of the data a larger sample size is needed. Second, more comprehensive clinical information on race, living environment, and family history is needed. Third, the current study is a single cohort study which could have led to bias in the analysis. The findings in this study should be replicated and confirmed in other populations. Finally, the underlying molecular mechanism of NDC80 complex in the process of tumorigenesis was not studied. Hence, for better understanding, NDC80 complex and its signal transduction pathway need to be further studied. Although there are a large number of studies on the role of the NDC80 complex genes in cancer, this study has for the first time developed a risk assessment score by including clinical factors and expression of the NDC80 complex with diagnostic and prognostic value in LUAD.

## Conclusions

In this study, it was found that NDC80, NUF2, SPC24 and SPC25 genes were differentially expressed in tumor tissues and normal tissues and NDC80, NUF2, SPC24 and SPC25genes have diagnostic values for LUAD. The combination of these genes also have diagnostic value for LUAD and have an advantage over NDC80, NUF2, SPC24 and SPC25 alone with regard to LUAD diagnosis. Validation of the prognostic value of NDC80 complex gene indicated that NDC80 and SPC25 were correlated with the prognosis of LUAD. Furthermore, high expression of NDC80, NUF2, SPC24 and SPC25 was associated with poor OS in Univariate survival. High expression level of NDC80 and SPC25 was related to poor OS in multivariate survival analysis. High expression of NDC80 combined with high expression of SPC25in LUAD was related to poor OS in joint analysis. Although we are evaluating the possible mechanism of the NDC80 complex genes in LUAD OS using GSEA, DAVID, etc., has established a nomogram to diagnose and predict LUAD. NDC80 complex gene is expected to be an indicator of early diagnosis and prognosis of LUAD. The combined detection of NDC80, NUF2, SPC24 and SPC25 may become a new research direction in tumor diagnosis and a new target for tumor targeted gene therapy. But these results require further verification in the next study.

## Supplementary Material

Supplementary tables.

## Figures and Tables

**Table 1:** Demographic and clinical data for 500 LUAD patients

Variables	Patients (n=500)	No.of events (%)	MST (days)	HR (95% CI)	Log-rank *P*
**Sex**					
Male	230	86(37.4%)	1528	Ref. 0.955(0.713-1.278)	0.755
Female	270	96(35.6%)	1454		
Missing	0	-	-	-	-
**Neoplasm status**					
With Tumor	164	112(68.3%)	864	Ref. 0.161(0.111-0.233)	<0.001
Tumor Free	284	37(13.0%)	4961		
Missing	52	-	-	-	-
**Stage**					
I	268	65(24.3%)	2620	Ref. 2.472(1.718-3.557) 3.495(2.383-5.126) 3.819(2.201-6.629)	<0.001
II	119	54(45.4%)	1209		
III	80	46(56.2%)	879		
IV	25	16(64%)	826		
Missing	8	-	-	-	-
**Residual tumor**					
Yes	335	128(38.4%)	1516	Ref. 4.029(2.247-7.222)	<0.001
No	16	13(81.2%)	464		
Missing	149	-	-	-	-
**Anatomic neoplasm subdivision**					
Left	192	71(37.0%)	1600	Ref. 1.035(0.766-1.399)	0.821
Right	296	106(35.8%)	1454		
Missing	12	-	-	-	-
**Targeted molecular therapy**					
Yes	149	54(36.2%)	1293	Ref. 0.832(0.598-1.158)	0.275
No	297	108(36.4%)	1600		
Missing	54	-	-	-	-
**Radiation therapy**					
Yes	60	35(58.3%)	896	Ref. 0.484(0.332—0.704)	<0.001
No	388	127(32.7%)	1632		
Missing	52	-	-	-	-
**Smoking**					
Positive	71	27(38%)	1421	Ref. 0.881(0.583-1.330)	0.546
Negative	415	145(34.9%)	1501		
Missing	14	-	-	-	-

MST: median survival time; HR: hazard ratio; CI: confidence interval.

**Table 2:** Univariate and multivariate survival analyses

Gene	Patients (n=500)	No. of events (%)	MST (days)	Crude HR (95% CI)	Crude *P*	Adjusted HR* (95% CI)	Adjusted P*
**NDC80**							
High	250	106(42.4%)	1288	Ref. 0.661 (0.492-0.888)	0.006	Ref. 0.635 (0.419-0.962)	0.032
Low	250	76(30.4%)	1600				
Missing	0	-	-	-	-	-	-
**NUF2**							
High	250	107(42.8%)	1229	Ref. 0.654 (0.487-0.879)	0.005	Ref. 0.741 (0.493-1.114)	0.150
Low	250	75(30.0%)	1632				
Missing	0	-	-	-	-	-	-
**SPC24**							
High	250	106(42.4%)	1229	Ref. 0.662 (0.493-0.890)	0.006	Ref. 0.734 (0.490-1.099)	0.133
Low	250	76(30.4%)	1622				
Missing	0	-	-	-	-	-	-
**SPC25**							
High	250	108(43.2%)	1171	Ref. 0.609 (0.453-0.819)	0.001	Ref. 0.657 (0.434-0.995)	0.047
Low	250	74(29.6%)	1632				
Missing	0	-	-	-	-	-	-

**Notes:** *adjustment for radiation therapy history; targeted therapy history; neoplasm status; TNM stage and residual tumors. **Abbreviations:** MST, median survival time; HR, hazard ratio; CI, confidence interval.

**Table 3:** Grouping according to expression levels of the SPC25 and NDC80 gene

Group	Composition
I	Low SPC25 + Low NDC80
II	Low SPC25 + High NDC80
	High SPC25 + Low NDC80
III	High SPC25 + High NDC80

**Table 4:** Joint-effects survival analysis

Group	Patients (n=500)	MST (days)	Crude *P*	Crude HR (95% CI)	Adjusted P*	Adjusted HR* (95% CI)
I	219	1632	0.004	Ref.	0.039	Ref.
II	62	1492	0.050	1.582(1.000-2.503)	0.064	1.799(0.966-3.351)
III	219	1171	0.001	1.698(1.232-2.341)	0.017	1.732(1.102-2.722)

**Notes:** *adjustment for radiation therapy history; targeted therapy history; neoplasm status; TNM stage and residual tumors. Abbreviations: MST, median survival time; HR, hazard ratio; CI, confidence interval.

## References

[1] Bray F, Ferlay J, Soerjomataram I, Siegel RL, Torre LA, Jemal A (2018). Global cancer statistics 2018: GLOBOCAN estimates of incidence and mortality worldwide for 36 cancers in 185 countries. CA: a cancer journal for clinicians.

[2] Siegel RL, Miller KD, Jemal A (2019). Cancer statistics, 2019. CA: a cancer journal for clinicians.

[3] Coudray N, Ocampo PS, Sakellaropoulos T, Narula N, Snuderl M, Fenyo D (2018). Classification and mutation prediction from non-small cell lung cancer histopathology images using deep learning. Nat Med.

[4] Torre LA, Bray F, Siegel RL, Ferlay J, Lortet-Tieulent J, Jemal A (2015). Global cancer statistics, 2012. CA: a cancer journal for clinicians.

[5] Chen Z, Fillmore CM, Hammerman PS, Kim CF, Wong KK (2014). Non-small-cell lung cancers: a heterogeneous set of diseases. Nature reviews Cancer.

[6] Liao Y, Yin G, Wang X, Zhong P, Fan X, Huang C (2019). Identification of candidate genes associated with the pathogenesis of small cell lung cancer via integrated bioinformatics analysis. Oncol Lett.

[7] Li B, Cui Y, Diehn M, Li R (2017). Development and Validation of an Individualized Immune Prognostic Signature in Early-Stage Nonsquamous Non-Small Cell Lung Cancer. JAMA oncology.

[8] Chen J, Chen H, Yang H, Dai H (2018). SPC25 upregulation increases cancer stem cell properties in non-small cell lung adenocarcinoma cells and independently predicts poor survival. Biomed Pharmacother.

[9] Cheeseman IM, Chappie JS, Wilson-Kubalek EM, Desai A (2006). The conserved KMN network constitutes the core microtubule-binding site of the kinetochore. Cell.

[10] DeLuca JG, Dong Y, Hergert P, Strauss J, Hickey JM, Salmon ED (2005). Hec1 and nuf2 are core components of the kinetochore outer plate essential for organizing microtubule attachment sites. Molecular biology of the cell.

[11] Janczyk PL, Skorupka KA, Tooley JG, Matson DR, Kestner CA, West T (2017). Mechanism of Ska Recruitment by Ndc80 Complexes to Kinetochores. Developmental cell.

[12] Kudalkar EM, Scarborough EA, Umbreit NT, Zelter A, Gestaut DR, Riffle M (2015). Regulation of outer kinetochore Ndc80 complex-based microtubule attachments by the central kinetochore Mis12/MIND complex. Proceedings of the National Academy of Sciences of the United States of America.

[13] Powers AF, Franck AD, Gestaut DR, Cooper J, Gracyzk B, Wei RR (2009). The Ndc80 kinetochore complex forms load-bearing attachments to dynamic microtubule tips via biased diffusion. Cell.

[14] Ju LL, Chen L, Li JH, Wang YF, Lu RJ, Bian ZL (2017). Effect of NDC80 in human hepatocellular carcinoma. World journal of gastroenterology.

[15] Zhao G, Oztan A, Ye Y, Schwarz TL (2019). Kinetochore Proteins Have a Post-Mitotic Function in Neurodevelopment. Developmental cell.

[16] Tang Z, Li C, Kang B, Gao G, Li C, Zhang Z (2017). GEPIA: a web server for cancer and normal gene expression profiling and interactive analyses. Nucleic acids research.

[17] Huang da W, Sherman BT, Lempicki RA (2009). Systematic and integrative analysis of large gene lists using DAVID bioinformatics resources. Nature protocols.

[18] Huang da W, Sherman BT, Lempicki RA (2009). Bioinformatics enrichment tools: paths toward the comprehensive functional analysis of large gene lists. Nucleic acids research.

[19] Maere S, Heymans K, Kuiper M (2005). BiNGO: a Cytoscape plugin to assess overrepresentation of gene ontology categories in biological networks. Bioinformatics (Oxford, England).

[20] Warde-Farley D, Donaldson SL, Comes O, Zuberi K, Badrawi R, Chao P (2010). The GeneMANIA prediction server: biological network integration for gene prioritization and predicting gene function. Nucleic acids research.

[21] Szklarczyk D, Franceschini A, Wyder S, Forslund K, Heller D, Huerta-Cepas J (2015). STRING v10: protein-protein interaction networks, integrated over the tree of life. Nucleic acids research.

[22] Lossos IS, Czerwinski DK, Alizadeh AA, Wechser MA, Tibshirani R, Botstein D (2004). Prediction of survival in diffuse large-B-cell lymphoma based on the expression of six genes. The New England journal of medicine.

[23] Alizadeh AA, Gentles AJ, Alencar AJ, Liu CL, Kohrt HE, Houot R (2011). Prediction of survival in diffuse large B-cell lymphoma based on the expression of 2 genes reflecting tumor and microenvironment. Blood.

[24] Balachandran VP, Gonen M, Smith JJ, DeMatteo RP (2015). Nomograms in oncology: more than meets the eye. The Lancet Oncology.

[25] Subramanian A, Tamayo P, Mootha VK, Mukherjee S, Ebert BL, Gillette MA (2005). Gene set enrichment analysis: a knowledge-based approach for interpreting genome-wide expression profiles. Proceedings of the National Academy of Sciences of the United States of America.

[26] Alushin GM, Lander GC, Kellogg EH, Zhang R, Baker D, Nogales E (2014). High-resolution microtubule structures reveal the structural transitions in alphabeta-tubulin upon GTP hydrolysis. Cell.

[27] Wilson-Kubalek EM, Cheeseman IM, Milligan RA (2016). Structural comparison of the Caenorhabditis elegans and human Ndc80 complexes bound to microtubules reveals distinct binding behavior. Molecular biology of the cell.

[28] Alushin GM, Ramey VH, Pasqualato S, Ball DA, Grigorieff N, Musacchio A (2010). The Ndc80 kinetochore complex forms oligomeric arrays along microtubules. Nature.

[29] Alushin GM, Musinipally V, Matson D, Tooley J, Stukenberg PT, Nogales E (2012). Multimodal microtubule binding by the Ndc80 kinetochore complex. Nature structural & molecular biology.

[30] Tang NH, Toda T (2015). MAPping the Ndc80 loop in cancer: A possible link between Ndc80/Hec1 overproduction and cancer formation. Bioessays.

[31] Zhang G, Kelstrup CD, Hu XW, Kaas Hansen MJ, Singleton MR, Olsen JV (2012). The Ndc80 internal loop is required for recruitment of the Ska complex to establish end-on microtubule attachment to kinetochores. J Cell Sci.

[32] Tang NH, Toda T (2013). Ndc80 Loop as a protein-protein interaction motif. Cell division.

[33] Zhao G, Cheng Y, Gui P, Cui M, Liu W, Wang W (2019). Dynamic acetylation of the kinetochore-associated protein HEC1 ensures accurate microtubule-kinetochore attachment. The Journal of biological chemistry.

[34] Shin J, Jeong G, Park JY, Kim H, Lee I (2018). MUN (MERISTEM UNSTRUCTURED), encoding a SPC24 homolog of NDC80 kinetochore complex, affects development through cell division in Arabidopsis thaliana. Plant J.

[35] Suzuki A, Badger BL, Haase J, Ohashi T, Erickson HP, Salmon ED (2016). How the kinetochore couples microtubule force and centromere stretch to move chromosomes. Nat Cell Biol.

[36] Diaz-Rodriguez E, Sotillo R, Schvartzman JM, Benezra R (2008). Hec1 overexpression hyperactivates the mitotic checkpoint and induces tumor formation in vivo. Proceedings of the National Academy of Sciences of the United States of America.

[37] Sotillo R, Hernando E, Diaz-Rodriguez E, Teruya-Feldstein J, Cordon-Cardo C, Lowe SW (2007). Mad2 overexpression promotes aneuploidy and tumorigenesis in mice. Cancer cell.

[38] Qu Y, Li J, Cai Q, Liu B (2014). Hec1/Ndc80 is overexpressed in human gastric cancer and regulates cell growth. Journal of gastroenterology.

[39] Meng QC, Wang HC, Song ZL, Shan ZZ, Yuan Z, Zheng Q (2015). Overexpression of NDC80 is correlated with prognosis of pancreatic cancer and regulates cell proliferation. American journal of cancer research.

[40] Xu B, Wu DP, Xie RT, Liu LG, Yan XB (2017). Elevated NDC80 expression is associated with poor prognosis in osteosarcoma patients. European review for medical and pharmacological sciences.

[41] Xing XK, Wu HY, Chen HL, Feng HG (2016). NDC80 promotes proliferation and metastasis of colon cancer cells.

[42] Zhang T, Zhou Y, Qi ST, Wang ZB, Qian WP, Ouyang YC (2015). Nuf2 is required for chromosome segregation during mouse oocyte meiotic maturation. Cell cycle (Georgetown, Tex).

[43] Liu Q, Dai SJ, Li H, Dong L, Peng YP (2014). Silencing of NUF2 inhibits tumor growth and induces apoptosis in human hepatocellular carcinomas. Asian Pacific journal of cancer prevention: APJCP.

[44] Zhou J, Yu Y, Pei Y, Cao C, Ding C, Wang D (2017). A potential prognostic biomarker SPC24 promotes tumorigenesis and metastasis in lung cancer. Oncotarget.

[45] McCleland ML, Kallio MJ, Barrett-Wilt GA, Kestner CA, Shabanowitz J, Hunt DF (2004). The vertebrate Ndc80 complex contains Spc24 and Spc25 homologs, which are required to establish and maintain kinetochore-microtubule attachment. Current biology: CB.

[46] Zhu P, Jin J, Liao Y, Li J, Yu XZ, Liao W (2015). A novel prognostic biomarker SPC24 up-regulated in hepatocellular carcinoma. Oncotarget.

[47] Pathania R, Ramachandran S, Mariappan G, Thakur P, Shi H, Choi JH (2016). Combined Inhibition of DNMT and HDAC Blocks the Tumorigenicity of Cancer Stem-like Cells and Attenuates Mammary Tumor Growth. Cancer research.

